# Bufothionine inhibits colorectal cancer progression via the STAT3/p53/SLC7A11 signaling axis

**DOI:** 10.3389/fphar.2026.1885072

**Published:** 2026-07-30

**Authors:** Ruifang Xie, Nan Xiang, Gui Zhou, Zeye Tan, Yiming Feng, Lingru Chen, Xin Zhou

**Affiliations:** Department of Pharmacy, Longhua Hospital, Shanghai University of Traditional Chinese Medicine, Shanghai, China

**Keywords:** bufothionine, colorectal cancer, ferroptosis, network pharmacology, p53

## Abstract

**Background:**

Bufothionine, a major bioactive component of dried toad skin, has shown anti-tumor potential. However, its efficacy and underlying mechanisms in colorectal cancer (CRC) remain unclear.

**Objective:**

This study aimed to investigate the therapeutic potential of bufothionine and its underlying mechanisms against CRC.

**Methods:**

Anti-tumor efficacy was assessed using *in vitro* (CCK-8, migration assays) and *in vivo* (HT-29 xenograft) models. Network pharmacology, molecular docking and molecular dynamics simulations were employed to predict targets. Mechanisms were validated by biochemical assays, Western blot, IHC, and qPCR, with ferrostatin-1 applied to confirm ferroptosis specificity.

**Results:**

Bufothionine significantly inhibited HT-29 cell proliferation and migration. Integrated network pharmacology and computational modeling identified p53 and STAT3 as core targets, with potential interactions predicted between bufothionine and key ferroptosis regulators (including SLC7A11). *In vitro*, bufothionine induced ferroptosis, characterized by increased Fe^2+^, ROS, and MDA levels, and a decreased GSH/GSSG ratio; these effects were partially reversed by ferrostatin-1. *In vivo*, bufothionine reduced tumor weight indices, improved liver function by attenuating AST and ALP elevations without hepatosplenic toxicity, and regulated inflammatory cytokines. Mechanistically, bufothionine suppressed inflammatory activation (NLRP3, IL-1β), inhibited STAT3 phosphorylation, and modulated p53 activity, leading to the subsequent inhibition of the SLC7A11/GPX4 axis in both cells and tumor tissues.

**Conclusion:**

Bufothionine suppresses CRC by the STAT3/p53/SLC7A11 signaling axis in a manner associated with ferroptosis, suggesting its potential as a therapeutic agent for colorectal cancer.

## Graphical Abstract

**Figure:**
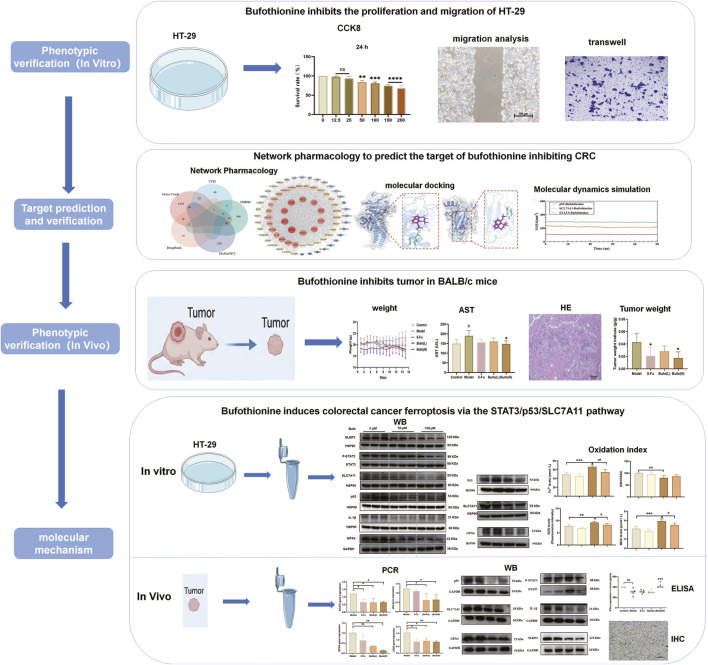

## 1 Introduction

Colorectal cancer (CRC) is the second leading cause of cancer-related deaths (Chen et al., 2020), with approximately 1.9 million new cases and nearly 900,000 CRC-associated deaths occurring annually (Sung et al., 2021; Siegel et al., 2022; Laterza et al., 2019). Notably, the incidence of CRC has a concerning rise among adults under 50 years of age in recent years (Siegel et al., 2024). Known risk factors for CRC development include demographic factors (such as age and gender), behavioral factors (e.g., smoking, physical inactivity, and obesity), lifestyle patterns, chronic psychological stress, and environmental exposures (Laterza et al., 2019; Wei et al., 2021). In particular, modern dietary patterns characterized by low fiber intake and high fat intake have been closely associated with the increased prevalence of CRC (Wang et al., 2021; Cross et al., 2010).

Conventional treatment modalities for colorectal cancer (CRC) mainly involve surgery, chemotherapy, radiotherapy, and targeted therapy (Cheng et al., 2018; Song et al., 2023). Although surgical resection remains the most effective intervention, many patients are diagnosed at advanced stages, precluding the opportunity for curative surgery (Ni et al., 2022). While chemotherapy provides clinical benefit, it is often accompanied by systemic toxicity and side effects that limit its clinical utility (Yu et al., 2019). Thus, there is a pressing need to identify more effective and tolerable therapeutic agents for CRC.

Ferroptosis, a form of regulated cell death driven by iron-dependent lipid peroxidation, has emerged as a promising target for cancer therapy (Dixon et al., 2012). Its distinct mechanism, which involves the accumulation of lethal lipid reactive oxygen species (ROS) and glutathione (GSH) biosynthesis, differs from other forms of cell death such as apoptosis (Dixon and Olzmann, 2024; Zhou et al., 2024). Growing evidence indicates that ferroptosis plays a significant role in tumor biology, and its induction can suppress cancer progression (Luo et al., 2024; Zhu and Du, 2024). Notably, some natural products have been reported to exert antitumor effects by inducing ferroptosis (Wang M. et al., 2024). Therefore, the induction of ferroptosis provides a new strategy for CRC treatment.

Bufothionine, an indole alkaloid compound, is extracted from *Bufo bufo gargarizans* Cantor or *Bufo melanostictus* Schneider. Its clinical effects and the associated mechanisms have been progressively demonstrated in recent years. It is recognized as a major bioactive ingredient with relatively high blood drug exposure in cinobufacini injection (a standardized aqueous extract preparation derived from dried toad skin) (Li et al., 2022), which is​ a clinically used formulation that is widely applied in the treatment of various tumors, including colorectal cancer (CRC) (Liu et al., 2022; Zhang et al., 2018; Wang M. et al., 2024). Although previous studies have showed that bufothionine exerts anti-inflammatory effects possibly by inducing neutrophil apoptosis (Zhang et al., 2021). In addition, it also exhibits anti-tumor activity in hepatocellular carcinoma models through suppressing proliferation and modulating autophagy and apoptosis (Xie et al., 2012; Kong et al., 2021; Xie et al., 2015). However, the underlying mechanisms of bufothionine in the treatment of CRC remain incompletely understood.

This study was designed to systematically investigate the anti-CRC activity of bufothionine and elucidate its underlying mechanisms. We first evaluated its effects on proliferation and migration in HT-29 cells. Then, network pharmacology, molecular docking, and molecular dynamics simulations were employed to predict its potential targets and signaling pathways. Finally, the antitumor efficacy and mechanisms were further assessed in a xenograft mouse model. This work provides new insights into the molecular mechanism of bufothionine against CRC and supports its potential as a candidate agent for CRC therapy.

## 2 Materials and methods

### 2.1 Chemicals and reagents

Bufothionine (HS21311B1, 98% purity) was obtained from Baoji Herbest Bio-Tech Co., Ltd. (China). Dimethyl sulfoxide (DMSO) was procured from Sigma-Aldrich. 5-Fluorouracil (5-Fu, batch no. M17GS148678) was supplied by Shanghai Yuanye Bio-Technology Co., Ltd. (China).

Cytokine assay kits for IFN-γ (YX-E11382), IL-1β (YX-E20533), IL-4 (YX-E20011), IL-6 (YX-E20012), IL-10 (YX-E20005), TNF-α (YX-E20220), and TGF-β (YX-E20217) were obtained from Sinobestbio (Shanghai, China). Biochemical assay kits for GSH (YX-W-A200), SOD (YX-W-A500), and ROS (YX-W-ROS) were also acquired from Sinobestbio. MDA (2508005001) was supplied by Shanghai solarbio Bioscience & Technology Co., LTD. Ferrostatin-1 (Fer-1, #374576), Necrostatin-1 (Nec-1, #422075), and MCC950 (#778137) were bought from MedChemExpress (MCE).

The following antibodies were used in this study: anti-p-STAT3 (#94994S), anti-STAT3 (#12640S), anti-p53 (#2527S), and anti-NLRP3 (#15101S) from Cell Signaling Technology; anti-SLC7A11 (#1160150201) from Merck; anti-GPX4 (T56759F) from Abmart; anti-IL-1β (00098224) from Proteintech; HSP90 (GR3264797-4) from Abcam; anti-GAPDH (AC220730006), anti-Ki-67 (Gb151499), and anti-Cleaved-Caspase-3 (Gb11532) from Servicebio Technology Co., Ltd. (Wuhan, China).

### 2.2 Effect of bufothionine on CRC *in vitro*

#### 2.2.1 Cell culture

HT-29 cells (Human CRC cells) (Chinese Academy of Sciences) were cultured in DMEM medium containing 10% fetal bovine serum and 1% penicillin/streptomycin at 37 °C and 5% CO_2_ incubator.

#### 2.2.2 Cell viability assay

Cell viability was tested using the CCK-8 Kit. Cells were seeded in 96-well plates and treated as indicated. A CCK-8 working solution was then prepared at a 1:10 (v/v) ratio of reagent to culture medium. Subsequently, 100 µL of the working solution was added to each well, and the plates were incubated at 37 °C for 30 min. Absorbance was measured at 450 nm using a Multiskan FC microplate reader (Thermo Scientific, United States).

#### 2.2.3 Wound healing assay

HT-29 cells were seeded in 6-well culture plates and grown to 80% confluency. A 100 µL pipette tip was used to create a uniform scratch wound in each monolayer. After scratching, the detached cells were gently washed three times with PBS. The cells were then incubated in low-serum DMEM (containing 1% FBS) supplemented with bufothionine at concentrations of 0, 50, and 100 µM. Following 24 h of incubation, cell migration into the wound area was assessed by measuring the scratch width under an inverted microscope (×40 magnification), and images were captured for analysis.

#### 2.2.4 Transwell assay

HT-29 cells were re-suspended in medium containing 1% FBS at a density of 2 × 10^5^ cells/ml. A 200 μL aliquot of the cell suspension was seeded into the upper chamber of a Trans-well insert, while 600 μl of medium containing 10% FBS was added to the lower chamber. After cells attachment, the medium in the upper chamber was replaced with fresh 1% FBS medium containing the compound at indicated concentrations (0, 50, 100 μM). Following 24 h incubation, migrated cells on the lower membrane surface were fixed with 4% paraformaldehyde, stained with 0.2% crystal violet, and imaged under an inverted microscope (Olympus, United States). Migrated cells were quantified using ImageJ software.

### 2.3 Integrated network pharmacology prediction and computational validation of bufothionine’s anti-CRC targets

#### 2.3.1 Network pharmacology analysis

The potential targets of bufothionine were predicted by analyzing its chemical structure (obtained from PubChem) using the Swiss Target Prediction platform. CRC-related genes were systematically collected from multiple databases including Therapeutic Target Database (https://db.idrblab.net/ttd/), OMIM (https://omim.org/), Drug Bank (https://go.drugbank.com/), Dis GeNET (https://www.disgenet.org/), and Gene Cards (http://www.genecards.org/). Common targets were identified through Venn analysis and used to construct a PPI network via STRING database, with Cytoscape (v3.10.1) employed for network topology analysis.

Functional enrichment analysis of the overlapping targets was conducted using the DAVID database, with Gene Ontology (GO) and KEGG pathway analyses performed at a significance threshold of P < 0.01 (*Homo sapiens* background) (Kanehisa and Goto, 2000; Kanehisa, 2019; Kanehisa et al., 2023). The results were visualized to elucidate key biological processes and signaling pathways underlying bufothionine’s anti-CRC activity.

#### 2.3.2 Molecular docking

The 3D structure of the compound was downloaded from the TCMSP database (https://www.tcmsp-e.com/). The 3D structure of the target protein was downloaded from the PDB online platform (https://www.rcsb.org/), including the mutant p53 DNA-binding domain (R249S, PDB ID: 3D06), SLC7A11 (PDB ID: 7EPZ), and STAT3 (PDB ID: 6NJS).They were imported to Auto Dock Tool for a series of pretreatments, including hydrogenation, dehydration, etc. Then, the affinity between ligand and receptor was evaluated. The docking results were visualized and analyzed using PyMol software.

#### 2.3.3 Molecular dynamics simulation

Molecular dynamics (MD) simulations were performed using Gromacs 2022.03 to further validate the binding stability of bufothionine-target complexes identified through molecular docking. The protein-ligand system was prepared by converting PDB structures to GRO format, with ligand parameters generated using AmberTools 22 and Gaussian 16 W. The system underwent solvation (TIP3P water model), ionization, energy minimization, and equilibration (NVT/NPT) before conducting a 100 ns production run to analyze dynamic behavior (Sharma et al., 2021; Okumura, 2023).

The resulting trajectories were evaluated for key structural parameters, including Root Mean Square Deviation (RMSD), Root Mean Square Fluctuation (RMSF), Radius of Gyration (Rg), Solvent Accessible Surface Area (SASA), and intermolecular hydrogen bonds. Gibbs free energy was calculated based on RMSD and Rg values, while binding free energy was assessed using the MM/PBSA method to determine the stability of the protein-ligand complex. Energy decomposition analysis was performed to identify key residues contributing to the binding free energy.

### 2.4 *In vitro* verification experiment

#### 2.4.1 Cell viability assay with/without cell death inhibitors

To determine the involvement of ferroptosis, HT-29 cells were treated with bufothionine (100 µM) in the presence or absence of cell death inhibitors, including the ferroptosis inhibitor Fer-1 (1 µM), necroptosis inhibitor Nec-1 (30 µM), NLRP3 inflammasome inhibitor MCC950 (5 µM), and apoptosis inhibitor Z-VAD-FMK (20 µM), for 24 h. Cell viability was then measured by CCK-8 assay as described above.

#### 2.4.2 Western blotting

The cells were lysed with lysis buffer after drug treatment with or without Fer-1, and the protein concentration was determined using a BCA kit. The obtained proteins were loaded and separated by 10% SDS-PAGE, then transferred onto 0.22 μm PVDF membranes. The membranes were subsequently incubated with primary antibodies at 4 °C overnight. In this experiment, the determined proteins include p53 pathway-related ferroptosis proteins (p-STAT3, STAT3, p53, SLC7A11, GPX4) and inflammation-related proteins (NLRP3, IL-1β), with GAPDH and HSP90 serving as internal reference.

#### 2.4.3 Oxidative stress index and Fe^2+^ detection

Cells treated with or without Fer-1 were collected and homogenized to assess ferroptosis-related biochemical indices.

The concentration of Fe^2+^ was measured using an Iron Assay Kit (Sinobestbio, Shanghai, China) following the manufacturer’s protocol. A colorimetric approach was utilized for detection, with absorbance measured at 510 nm.

The levels of GSH, SOD, MDA, and ROS were quantified using commercially available assay kits (Sinobestbio, Shanghai, China). The GSH concentration was assessed at 412 nm, SOD activity at 560 nm, and MDA levels at 532 nm, all by spectrophotometry. ROS levels were analyzed using a fluorescence microplate reader (Thermo, Multiskan FC, United States) with excitation/emission wavelengths of 488 nm and 525 nm, respectively.

### 2.5 *In vivo* verification experiment

#### 2.5.1 Establishment of HT-29-tumor-bearing mice model

Male BALB/c nude mice (5–7 weeks old, SPF grade) were purchased from Shanghai SLAC Laboratory Animal Co., Ltd. and housed under standard conditions (temperature 18 °C–22 °C, humidity 65% ± 5%, 12 h light/dark cycle) with free access to food and water.

**FIGURE 1:**
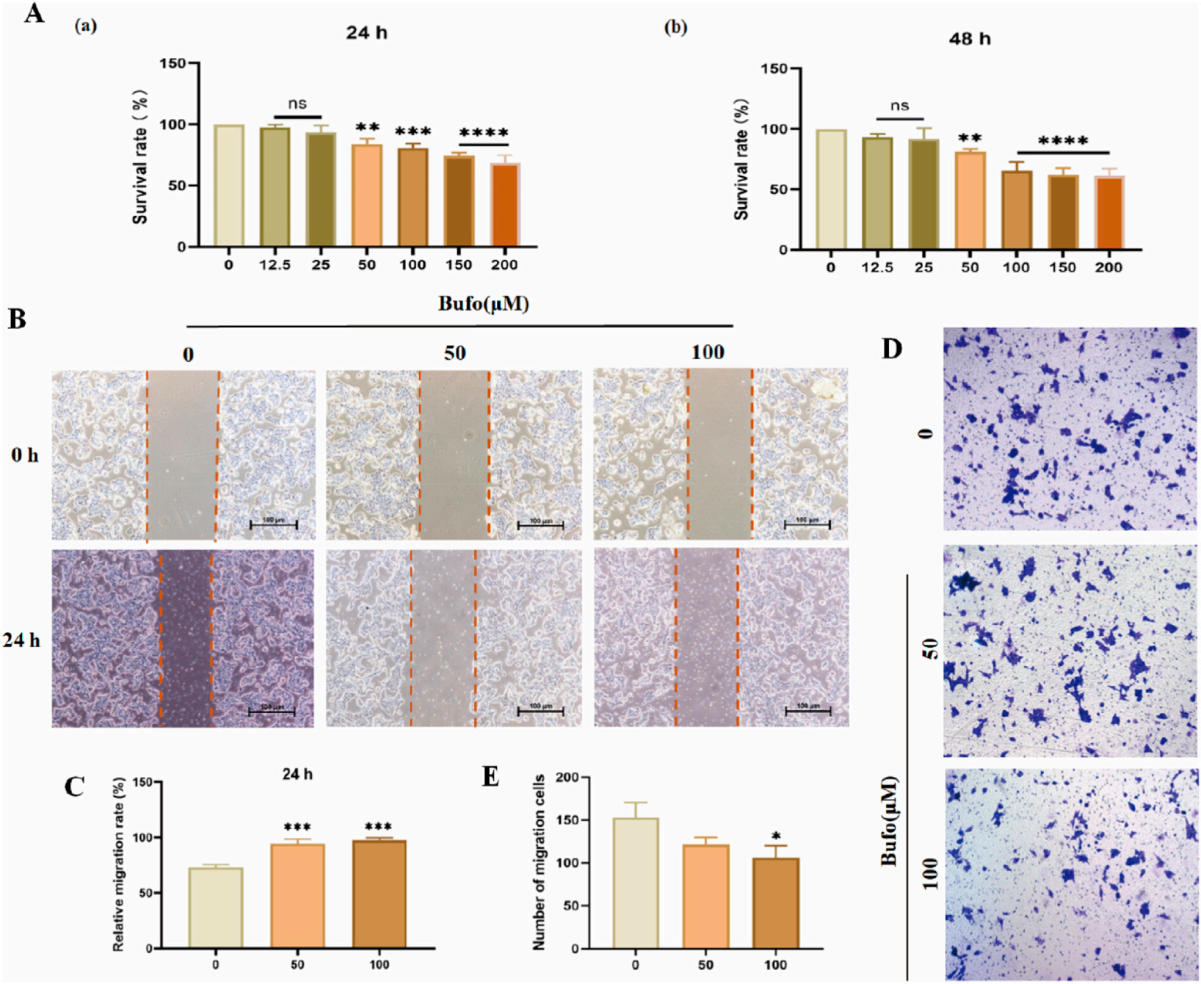
Effect of bufothionine on growth and migration of HT-29 cells. **(A)** HT-29 cells were treated with different concentrations of bufothionine for 24 h and 48 h, and cell survival rates were measured. **(B,C)** Wound-healing assays were used to assess the migratory ability of HT-29 cells exposed to bufothionine for 24 h **(D,E)** Transwell assays were performed to evaluate the effect of bufothionine on HT-29 cells migration. Data are represented as the mean ± SD. *P < 0.05, **P < 0.01, ***P < 0.001 vs. control (0 μM); ns: no significant difference.

They were allowed to adapt to the laboratory environment for a week prior to the experiment. HT-29 cells (1 × 10^8^ cells/mL in saline) were subcutaneously inoculated into the left axillary region of each mouse (0.2 mL per mouse). One week later, tumor-bearing mice were randomly divided into four groups: model (saline), positive control (5-Fu, 20 mg/kg, every other day), low-dose bufothionine (0.5445 mg/kg), and high-dose bufothionine (1.089 mg/kg) groups. A group of non-inoculated mice served as blank controls. The dosage determination was based on previous research (Xie et al., 2015). All treatments except 5-Fu were administered daily via intraperitoneal injection for 10 consecutive days (Figure 1A).

After the final treatment, mice were fasted for 16 h and euthanized. Blood was collected via orbital puncture, and serum was separated for cytokine and liver function analysis. Tumors were excised, weighed, and divided into two parts: one was snap-frozen for cytokine, PCR, and Western blot assays, and the other was fixed in formalin for histological processing and H&E staining. All procedures were supported by the National Animal Welfare Law of China and were performed according to the care guidelines of the Animal Ethics Committee of Shanghai University of TCM (LHERAW-22060).

#### 2.5.2 Hematoxylin and eosin (H&E) staining

The isolated tumor tissue was fixed in 4% paraformaldehyde and dehydrated using a graded alcohol series. Subsequently, the tissue was embedded in paraffin and sectioned into 7 μm-thick slices. The sections were then subjected to standard hematoxylin and eosin (H&E) staining and examined under a light microscope.

#### 2.5.3 Measurements of cytokines

The concentrations of IFN-γ, IL-1β, IL-4, IL-6, IL-10, TNF-α, and TGF-β in both tumor tissue and serum samples were quantitatively determined using commercially available enzyme-linked immunosorbent assay (ELISA) kits in accordance with the manufacturer’s protocols. The sample processing procedures were as follows: For tumor tissue analysis, samples were homogenized in lysis buffer and subsequently centrifuged at 15,000 rpm for 10 min at 4 °C. For serum preparation, the blood samples were collected and centrifuged at 3,000 rpm for 10 min at room temperature. The resulting supernatants from both tissue and serum preparations were collected and stored at −80 °C for further analysis.

#### 2.5.4 Immunohistochemistry (IHC)

Formalin-fixed, paraffin-embedded tissue sections of CRC animal model were sequentially processed through xylene for de-paraffinization and a graded alcohol series for rehydration. Endogenous peroxidase activity was quenched by treating the slides with 3% hydrogen peroxide in methanol for 10 min at room temperature. The samples were then incubated with primary antibodies specific to STAT3, p53, Ki-67, and cleaved caspase-3 at 4 °C overnight, followed by incubation with appropriate secondary antibodies for 1 h at room temperature. Between each incubation step, the slides were thoroughly washed 3 times with phosphate-buffered saline (PBS), with each wash lasting 5 min. Fluorescent images were captured using a confocal laser scanning microscope (Olympus, New York, United States).

#### 2.5.5 Biochemical assays in tumor tissues

The levels of Fe^2+^, GSH, SOD, MDA, and ROS in tumor tissues were measured using the same methods described above.

#### 2.5.6 Quantitative real-time PCR (qPCR)

Total RNA was isolated from the tumor tissue of CRC animal model using an RNA isolation kit (Servicebio, Wuhan, China) and reverse transcribed into cDNA using a reverse transcription kit (Servicebio, Wuhan, China) according to the supplier’s protocol. Gene expressions of STAT3,GPX4, p53 and COX2 in tumor tissue were quantified using a fluorescence q-PCR system (Bio-rad, United States) and were normalized to GAPDH mRNA levels. The specific primers information was shown in Table 1.

**TABLE 1:** Primer used for q-PCR.

Genes	Forward primer	Reverse primer	Fragment length (bp)
STAT3	TGC​GGA​GAA​GCA​TTG​TGA​GTG	TCT​TAA​TTT​GTT​GGC​GGG​TCT	210
COX2	GAA​ATA​TCA​GGT​CAT​TGG​TGG​AGA	ATG​CTC​CTG​CTT​GAG​TAT​GTC​G	205
GPX4	GCA​CAT​GGT​CTG​CCT​GGA​TAA​G	TCT​TGA​TTA​CTT​CCT​GGC​TCC​TG	192
p53	CCC​TCT​GAG​CCA​GGA​GAC​ATT	CCC​AGG​TGG​AAG​CCA​TAG​TTG	282
GAPDH	CCT​CGT​CCC​GTA​GAC​AAA​ATG	TGA​GGT​CAA​TGA​AGG​GGT​CGT	133

#### 2.5.7 Western blotting

Tumor tissues from the model, 5-Fu, and bufothionine-treated groups were homogenized, and total protein was extracted using RIPA lysis buffer. Protein concentrations were determined by BCA assay. Western blotting was performed as described above.

### 2.6 Statistical analysis

Statistical analysis was performed using GraphPad Prism software (version 10.4.1), with data presented as mean ± standard deviation (mean ± SD). For normally distributed data with homogeneous variance, comparisons between two groups were performed using the t-test, while comparisons among multiple groups were assessed using one-way ANOVA. When the data showed normally distributed data with heterogeneous variance, Welch’s ANOVA was applied. P value of less than 0.05 was applied to indicate statistical significance.

## 3 Results

### 3.1 Bufothionine inhibits HT-29 cell proliferation and migration *in vitro*

To evaluate the therapeutic potential of bufothionine against colorectal cancer (CRC), we assessed its effects on the proliferation and migration of HT-29 cells. CCK-8 assays (Figure 1A) revealed that bufothionine significantly suppressed cell growth in both concentration and time dependent manner, with notable inhibition observed at ≥50 μM (P < 0.01). Wound healing assays (Figures 1B,C) demonstrated the dose-dependent inhibition of cell migration at 24 h post-treatment. Consistent with these findings, Trans-well migration assays (Figures 1D,E) confirmed a dose-dependent impairment of HT-29 cell migration at 24 h, with statistical significance reached at 100 μM (P < 0.05), further supporting its anti-tumor activity. Collectively, these results indicated that bufothionine could exert potent anti-proliferative and anti-migratory effects on HT29 cells *in vitro*.

### 3.2 Computational prediction and validation of bufothionine’s anti-CRC targets

#### 3.2.1 Network pharmacology analysis results

**FIGURE 2:**
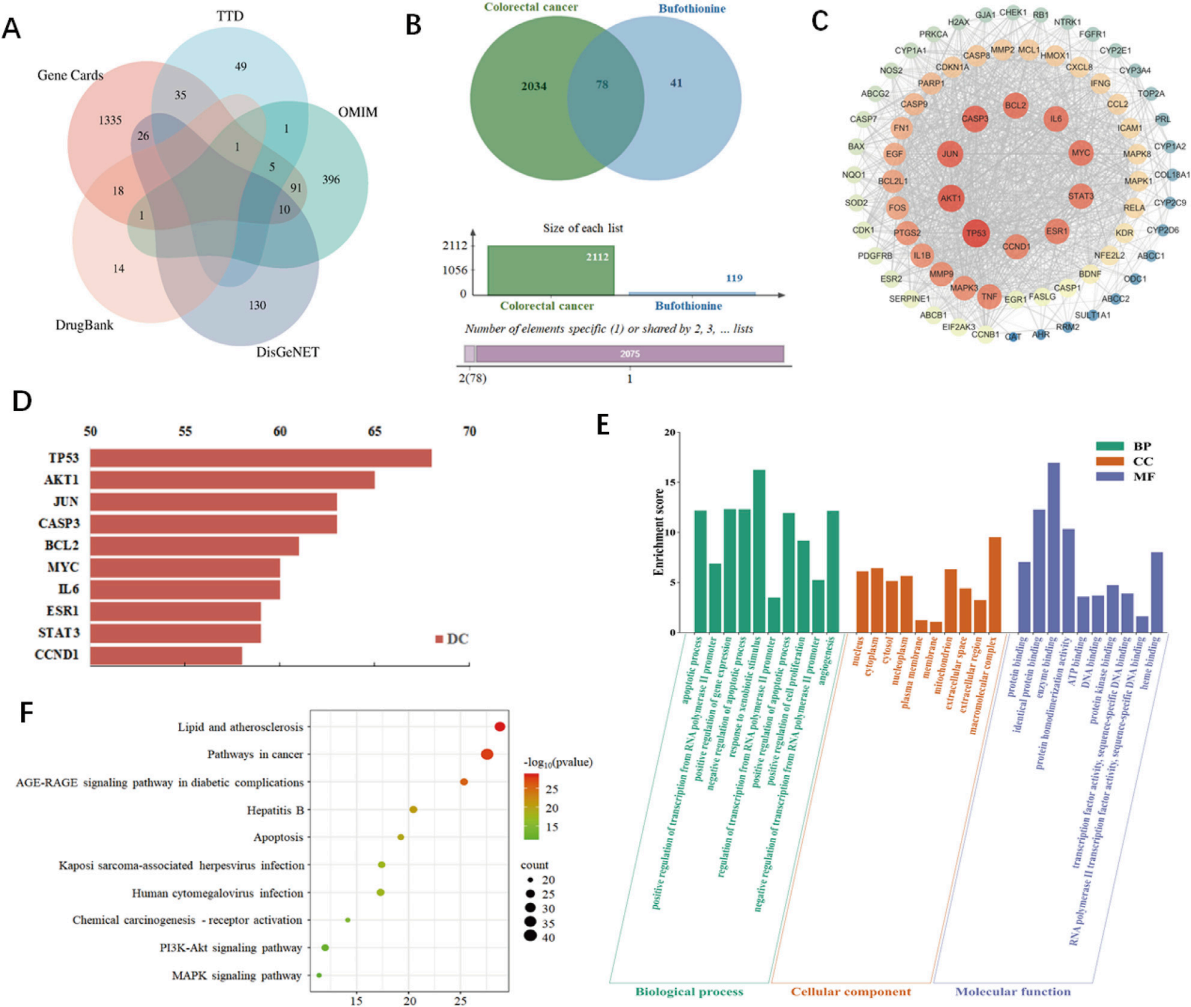
Analysis of the targets associated with bufothionine and CRC. **(A)** Venn diagram of CRC-related targets in five databases. **(B)** Venn diagram of bufothionine-CRC common targets. **(C)** PPI network of bufothionine on CRC. A tangerine color indicates higher relevance, lighter color less relevance and the core targets are in the inner circle. **(D)** Degree values of core anti-CRC targets in bufothionine. **(E)** GO enrichment analysis of bufothionine targets. Enrichment score (Y-axis), term (X-axis); The 10 core results of BP, CC, and MF are marked using green, orange, and purple, respectively. **(F)** KEGG pathway enrichment analysis of bufothionine targets. Pathways (Y-axis), FDR (X-axis), and P-value (color change). Bubble size means the number of genes enriched in the pathway (www.kegg.jp/kegg/kegg1.html).

A total of 2,112 CRC-related genes were collected from public databases (OMIM, DisGeNET, DrugBank, GeneCards, TTD), and 119 potential targets of bufothionine [C (N+)1(CCC2 = CNC3 = C2C1 = C(C=C3) OS(=O)O)C] were predicted using Swiss Target Prediction (Figure 2A). Venn analysis identified 78 overlapping targets (Figure 2B). Protein-protein interaction (PPI) network analysis of these 78 targets revealed 10 core proteins, with p53 exhibiting the highest connectivity (68 interactions), followed by AKT1, JUN, CASP3, BCL2, MYC, IL-6, ESR1, STAT3, and CCND1 (Figures 2C,D).

To investigate the molecular mechanisms of bufothionine’s anti-colorectal cancer activity, functional enrichment analyses were performed using the DAVID database. Gene Ontology (GO) analysis identified 622 significantly enriched terms, including 484 in biological process (BP), 46 in cellular component (CC), and 92 in molecular function (MF), respectively (Figure 2E). Kyoto Encyclopedia of Genes and Genomes (KEGG) pathway analysis revealed 162 significantly enriched pathways, with the most prominent being pathways in cancer and lipid metabolism/atherosclerosis (Figure 2F).

#### 3.2.2 Molecular docking analysis

**FIGURE 3:**
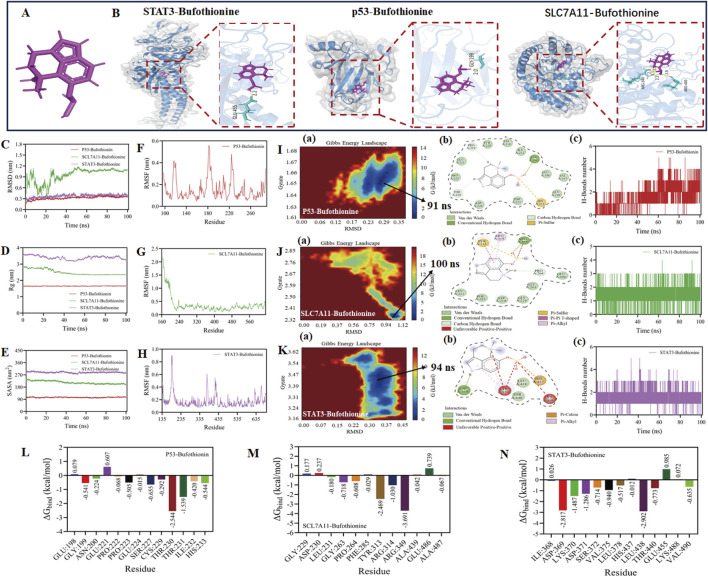
Molecular docking diagram and simulation analysis of bufothionine to targets. **(A)** Structure of bufothionine. **(B)** Molecular docking of bufothionine with STAT3, P53 and SLC7A11. **(C)** RMSD curves. **(D)** Rg curves. **(E)** SASA curves. RMSF curves of p53-Bufothionine **(F)**, SLC7A11-Bufothionine **(G)**, STAT3-Bufothionine **(H)**. Gibbs free energy analysis of p53-Bufothionine **(I)**, SCL7A11-Bufothionine **(J)**, STAT3-Bufothionine **(K)**. (a) 2D morphological plots of Gibbs free energy. (b) 2D interaction diagrams of three complexes at the lowest Gibbs energy moment. (c) 100ns-long hydrogen bonding changes of targets-bufothionine complexes. Amino acid binding free energy decomposition analysis of p53-Bufothionine **(L)**, SCL7A11-Bufothionine **(M)**, STAT3-Bufothionine **(N)**.

To further validate the key targets identified in the PPI network, molecular docking was performed to evaluate the binding affinity of bufothionine with CRC-related targets. Based on their central roles in the ferroptosis-related p53 pathway, p53, STAT3, and SLC7A11 were selected for analysis. Docking results showed that bufothionine exhibited favorable binding affinities with STAT3 (−6.774 kcal mol^-1^), p53 (−5.878 kcal mol^-1^), and SLC7A11 (−5.777 kcal mol^-1^), all below the common threshold of −5.0 kcal mol^-1^ (Figures 3A,B), suggesting potential ligand-target interactions. These findings suggest a potential regulatory association of bufothionine in the STAT3/p53/SLC7A11 signaling axis in colorectal cancer.

#### 3.2.3 Molecular dynamics simulation analysis

To validate the molecular docking results and investigate the dynamic binding characteristics, a 100-ns molecular dynamics (MD) simulation was performed. The system stability was comprehensively evaluated through multiple parameters, including RMSD, Rg, SASA, and RMSF.

The RMSD analysis, which reflects structural variations over time, demonstrated stable binding patterns for all three complexes (Figure 3C). p53-bufothionine and STAT3-bufothionine complexes both maintained consistent stability throughout the simulation, with RMSD values of mainly fluctuating within 0.25–0.40 nm and 0.30–0.45 nm, respectively. The SLC7A11-bufothionine complex showed slight fluctuations during the first 30 ns, after which the RMSD stabilized around 1.0–1.2 nm. Rg analysis, indicating the compactness of protein-ligand complexes, revealed that all three complexes maintained Rg values expected ranges (Figure 3D), with approximately 1.6–1.7 nm for p53, a decrease from ∼2.8 nm to ∼2.0 nm for SLC7A11, and 3.2–3.6 nm for STAT3, suggesting tight binding interactions. SASA analysis, reflecting protein surface accessibility, showed consistent profiles for all complexes during 100-ns simulation, with mean values of approximately 90–110 nm^2^ for p53, 200–230 nm^2^ for SLC7A11, and 270–300 nm^2^ for STAT3. (Figure 3E). RMSF analysis of residue flexibility identified key interaction regions: overall low flexibility for p53; localized fluctuations for STAT3; and elevated N-terminal flexibility with a stable core for SLC7A11 (Figures 3F–H).

Furthermore, thermodynamic analyses were performed to confirm binding stability. Gibbs free energy landscapes revealed multiple low-energy basins for the p53-bufothionine complex, a dominant low-energy basin for the SLC7A11-bufothionine complex, and a continuous low-energy region for the STAT3-bufothionine complex (Figures 3Ia–Ka). Visualization of the lowest free-energy conformations showed that the p53-and STAT3-bufothionine complexes formed 1-5 hydrogen bonds, whereas the SLC7A11-bufothionine complex maintained 1-4 hydrogen bonds during the simulation (Figures 3Ib–Kb). MM/PBSA analysis based on the 100-ns trajectories yielded binding free energies of −16.18, −20.61, and −28.50 kcal/mol (Table 2). Finally, energy decomposition identified key contributing residues: eleven for p53 (e.g., THR:230, THR:231), nine for SLC7A11 (e.g., ARG:349, TYR:313), and ten for STAT3 (e.g., ASP369, LEU438) (Figures 3L–N).

**TABLE 2:** Complex binding free energy analysis (kcal/mol).

Energy contributions	p53-bufothionine	SLC7A11-bufothionine	STAT3-bufothionine
Δ*VDWAALS*	−26.90 ± 0.27	−25.85 ± 0.24	−34.90 ± 0.17
Δ*E*_*elec*_	−31.06 ± 0.76	−42.29 ± 0.71	−50.21 ± 2.12
Δ*E*_*surf*_	−3.05 ± 0.01	−3.45 ± 0.07	−4.06 ± 0.09
Δ*G*_*gas*_	−57.96 ± 0.80	−68.15 ± 0.75	−85.10 ± 2.12
Δ*G*_*solvation*_	41.78 ± 0.05	47.54 ± 1.78	56.60 ± 3.14
Δ*G*_*Bind*_	−16.18 ± 0.8	−20.61 ± 1.93	−28.50 ± 3.79

Δ*VDWAALS*: van der waals force; Δ*E*_*elec*_: electrostatic force; Δ*E*_*surf*_: solvent accessible surface area; Δ*G*_*gas*_: gas-phase energy; Δ*G*_*solvation*_: solvation free energy; Δ*G*_*Bind*_: total binding free energy.

### 3.3 Effect and mechanism of bufothionine on CRC *in vitro*

**FIGURE 4:**
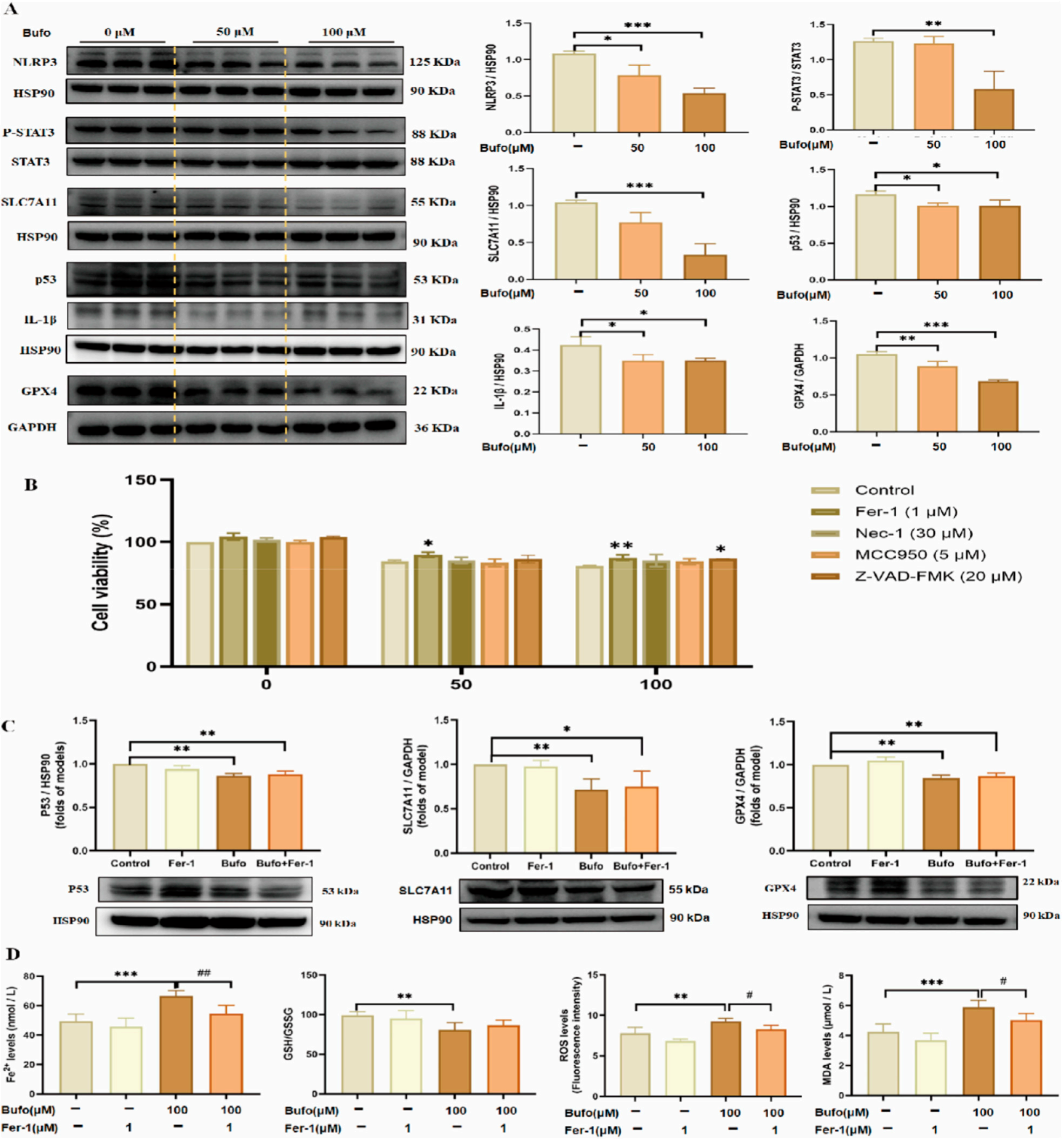
Bufothionine induces ferroptosis in HT-29 cells. **(A)** Western blot analysis of proteins related to inflammation (NLRP3, IL-1β, STAT3) and ferroptosis (SLC7A11, GPX4, p53). (n = 3). **(B)** HT-29 cells were treated with bufothionine (50, 100 μM) in the presence or absence of Fer-1 (1 μM), Nec-1 (30 μM), MCC950 (5 μM), Z-VAD-FMK (20 μM). Cell viability was detected by CCK8 assay. **(C)** Western blot analysis of key ferroptosis-related proteins (p53, SLC7A11, GPX4) in the presence or absence of Fer-1 (1 μM). **(D)** The levels of Fe^2+^, GSH/GSSG ratio, ROS, and MDA were determined. Data are presented as mean ± SD (n = 3). *P < 0.05, **P < 0.01, ***P < 0.001 vs. control group; #P < 0.05, ##P < 0.01 vs. bufothionine-treated group (without Fer-1).

To investigate whether bufothionine induces ferroptotic cell death in HT-29 cells, we assessed canonical ferroptosis hallmarks and the p53-mediated regulatory pathway. As shown in Figure 4A, bufothionine treatment significantly downregulated the levels of p-STAT3, SLC7A11, GPX4, NLRP3, p53 and IL-1β in a dose-dependent manner compared to the control group (P < 0.05).

To further evaluate the role of ferroptosis, CCK-8 assays were performed to determine whether specific cell inhibitors could reverse bufothionine-induced cytotoxicity in HT-29 cells following 24 h bufothionine exposure. As shown in Figure 4B, the inhibition of cell viability caused by bufothionine was significantly reversed by ferroptosis inhibitors (Fer-1) and apoptosis inhibitors (Z-VAD-FMK) in a dose-dependent manner, indicating that both ferroptosis and apoptosis may be involved in bufothionine-induced cytotoxicity. Biochemical assays (Figure 4D) revealed that bufothionine significantly increased Fe^2+^, ROS, and MDA levels (P < 0.01), and decreased the GSH/GSSG ratio (P < 0.01). Co-treatment with Fer-1 significantly attenuated the increases in Fe^2+^ (P < 0.01), ROS (P < 0.05), and MDA (P < 0.05), and partially reversed the decrease in the GSH/GSSG ratio. Corresponding Western blot analysis (Figure 4C) showed that Fer-1 also partially reversed bufothionine-induced downregulation of p53, SLC7A11 and GPX4, which may reflect a feedback regulation of the oxidative stress initiated by ferroptosis.

Collectively, these results demonstrated that bufothionine could induce ferroptosis in HT-29 cells primarily through p53 downregulation and subsequent oxidative stress, and this process could be rescued by the ferroptosis inhibitor Fer-1.

### 3.4 Effect and mechanism of bufothionine on CRC *in vivo*

#### 3.4.1 Antitumor efficacy and systemic safety of bufothionine in a xenograft model

**FIGURE 5:**
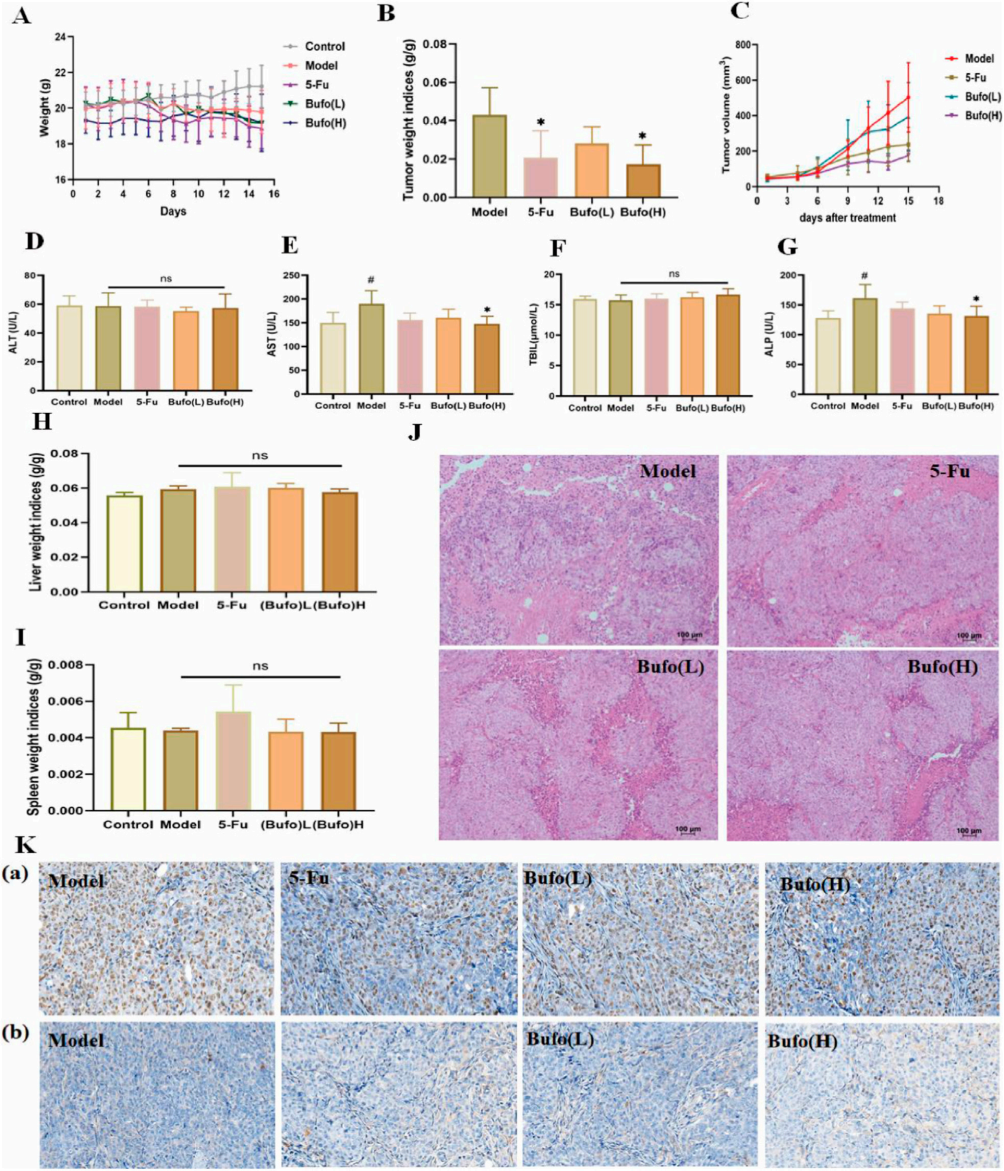
Effect of bufothionine on HT29-induced transplanted tumor in nude mice. **(A)** Body weight. **(B)** Tumor weight indices. **(C)** Tumor volume growth curves over time. **(D)** ALT. **(E)** AST. **(F)** TBIL. **(G)** ALP. **(H)** Liver weight indices. **(I)** Spleen weight indices. **(J)** Representative H&E staining images of tumor tissue (100×). **(K)** Immunohistochemical staining of tumor tissues for Ki-67 (a) and cleaved caspase-3 (b) (400×).​ Data are represented as mean ± SD, n = 5.

In the HT-29 xenograft model, tumor weight indices (Figure 5B) were significantly lower in the Bufo (H) group and the 5-Fu group compared with the model group (P < 0.05). Analysis of tumor volume growth curves (Figure 5C) revealed distinct progression patterns: the model group showed the most rapid tumor growth, while Bufo(L) and 5-Fu showed intermediate growth, and Bufo(H) showed the most pronounced inhibition of tumor growth. Histopathological analysis of tumor tissues (Figure 5J) revealed that both bufothionine and 5-Fu alleviated pathological morphological changes, consistent with their antitumor activity.

To further assess the antitumor effect, tumor tissues were stained for Ki-67 and cleaved caspase-3. Compared with the model group, bufothionine treatment reduced Ki-67 expression and increased cleaved caspase-3 expression (Figure 5K), indicating that bufothionine may inhibit tumor cell proliferation and is associated with increased apoptotic cell death.

Regarding systemic safety, all groups exhibited weight loss following tumor inoculation. However, the body weight decline in bufothionine groups was more moderate compared with the 5-Fu group (Figure 5A). Notably, mice in the 5-Fu group displayed clinical symptoms, including dull fur, poor spirit and appetite, diarrhea, and hematochezia, while other groups were not observed, indicating that bufothionine induced fewer adverse reactions than 5-Fu. Liver and spleen weight indices (Figures 5H,I) showed no significant differences among groups, suggesting no obvious hepatosplenic toxicity. Furthermore, biochemical analysis (Figures 5D–G) revealed that serum ALT and TBIL levels were comparable across all groups, whereas bufothionine significantly reduced the elevated AST and ALP levels induced by tumor implantation, indicating a potential protective effect on liver function.

#### 3.4.2 Bufothionine modulates inflammatory cytokine profiles in serum and tumor tissue

Inflammatory cytokines are key mediators of the tumor microenvironment, critically influencing tumor progression and immune response. Our results demonstrated that bufothionine treatment could significantly modulate the expression of these pivotal cytokines.

**FIGURE 6:**
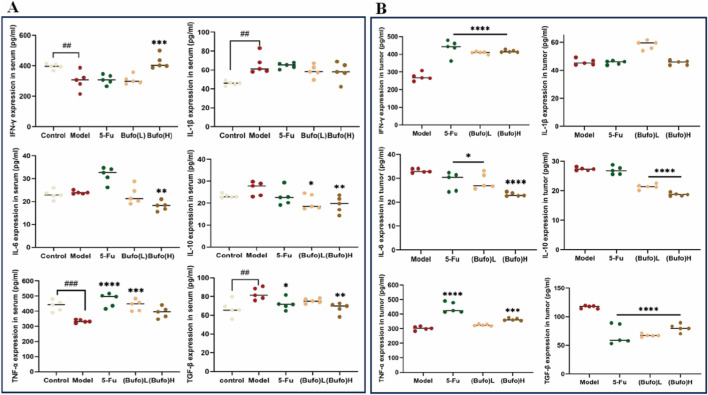
Effect of bufothionine on cytokines in serum and tumor tissue. The contents of IFN-γ, IL-1β, IL-6, IL-10, TGF-β, TNF-α in serum **(A)**, and tumor tissue **(B)** of each group by ELISA. Data are represented as the mean ± SD, n = 5. *P < 0.05, **P < 0.01, ***P < 0.001, ****P < 0.0001 vs. model group by ANOVA, #P < 0.05, ##P < 0.01, ###P < 0.001 vs. control group by ANOVA.

Serum ELISA results (Figure 6A) revealed that, compared with the vehicle group, the levels of IL-1β, IL-6, IL-10, and TGF-β in the model group showed an upregulatory trend, while IFN-γ and TNF-α were downregulated.​ Drug treatments suppressed the upregulation of IL-1β, IL-6, IL-10, and TGF-β, and reversed the downregulation of IFN-γ and TNF-α, with statistical significance observed in the Bufo (H) group for IFN-γ (P < 0.001), IL-6 (P < 0.01), IL-10 (P < 0.01), and TGF-β (P < 0.01).

Consistent regulatory effects were observed in tumor tissues (Figure 6B):​ bufothionine effectively downregulated IL-6, IL-10, and TGF-β, while upregulating IFN-γ and TNF-α. Most of these changes reached statistical significance in the Bufo(H) group, with P < 0.001 for IL-6, IL-10, TGF-β, IFN-γ, and TNF-α.

#### 3.4.3 Effect of bufothionine on CRC via p53-mediated ferroptosis pathway

**FIGURE 7:**
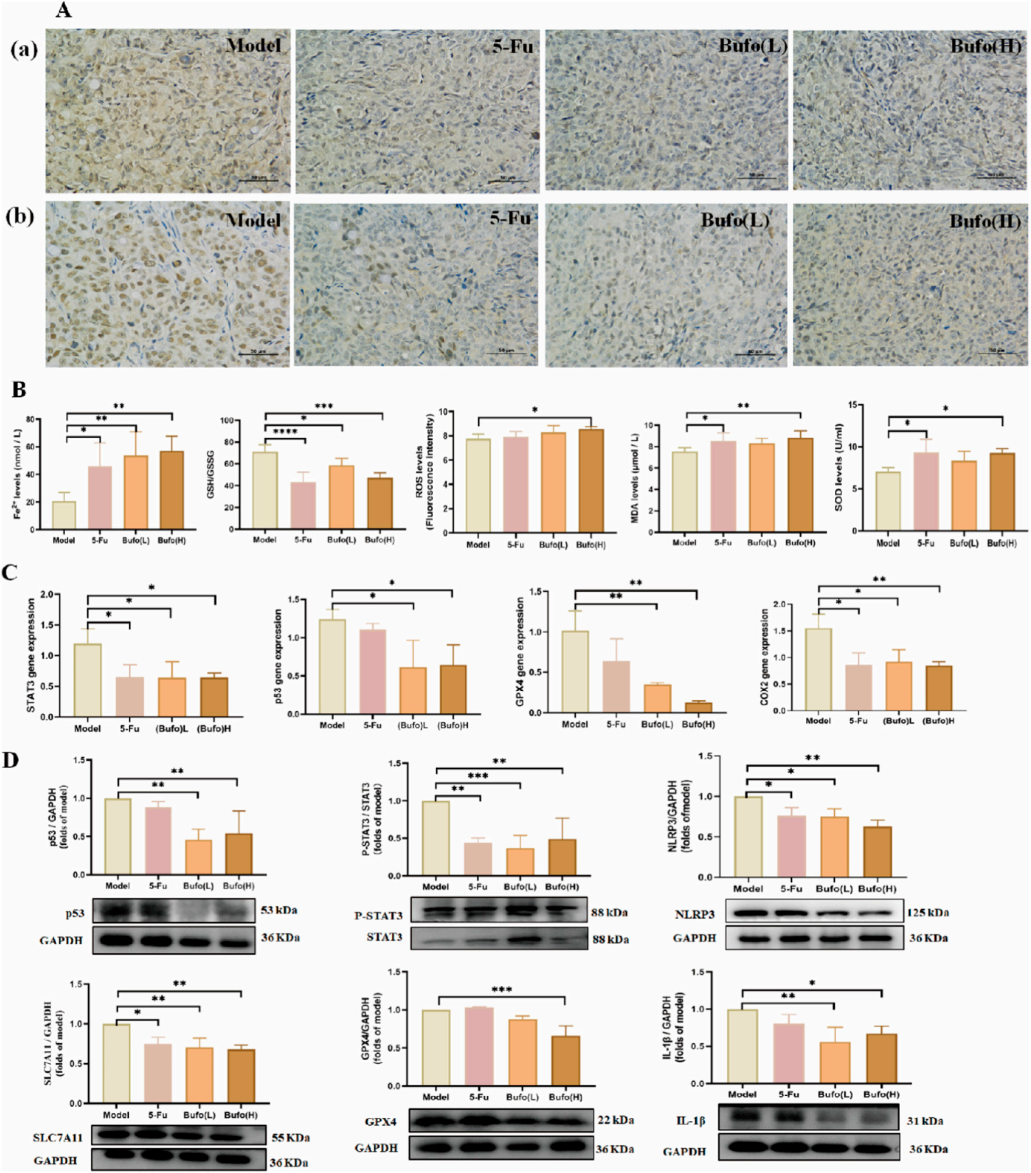
Effect of bufothionine on ferroptosis related indicators. **(A)** Representative IHC images of tumor tissue sections stained for STAT3 (a), and p53 (b) (400 ×). **(B)** Contents of Fe^2+^, GSH/GSSG, ROS, MDA, SOD in tumor tissues of each group (n = 5). **(C)** Gene expression of STAT3, p53, and GPX4 in tumor tissue of each group (n = 3). **(D)** Western blot analysis of the protein expression levels of p53, p-STAT3/STAT3, SLC7A11, GPX4, NLRP3, and IL-1β in tumor tissue (n = 3). *P < 0.05, **P < 0.01, ***P < 0.001, ****P < 0.0001 vs. model group by ANOVA.

To elucidate the mechanism, we investigated the p53-mediated ferroptosis pathway. Immunohistochemistry confirmed that bufothionine treatment decreased p53 and STAT3 protein expression in tumor tissues (Figure 7A).

Biochemical assays revealed (Figure 7B): compared to model group, bufothionine significantly elevate levels of Fe^2+^, ROS, SOD and MDA (P < 0.05), while significantly reducing the GSH/GSSG ratio (P < 0.001).

Q-PCR results demonstrated (Figure 7C): compared to the model group, bufothionine significantly downregulated the gene expressions of STAT3, p53, GPX4, and COX2 (P < 0.05) with the Bufo (H) group showing statistical significance (P < 0.001).

Western blotting results showed (Figure 7D): compared to the model group, bufothionine treatment significantly downregulated the protein expression of p-STAT3, p53, SLC7A11, GPX4, NLRP3, and IL-1β (P < 0.05).

## 4 Discussion

Ferroptosis, a regulated form of cell death, has emerged as a promising therapeutic strategy of colorectal cancer (CRC), a malignancy often characterized by altered redox homeostasis (Singh et al., 2025). CRC is marked by a high prevalence of TP53 mutations, which occur in approximately 60% of cases (Brannon et al., 2014; Xin et al., 2025).​ These mutations are predominantly missense and result in dual outcomes: the loss of wild-type tumor-suppressive function and, more importantly, the acquisition of oncogenic properties that promote tumor progression and cell survival (Liebl and Hofmann, 2021; Hu et al., 2021). In this study, we demonstrate that bufothionine, a principal bioactive alkaloid derived from clinically used dried toad skin and its preparation, exerts potent antitumor effects against CRC both *in vitro* and *in vivo*. Mechanistically, bufothionine modulates the STAT3/p53/SLC7A11 signaling axis, a pathway closely linked to ferroptosis regulation and mutant p53-mediated tumor progression.

The antitumor activity of bufothionine was initially evaluated *in vitro*. Treatment with bufothionine significantly and dose-dependently inhibited the proliferation and migration of HT-29 cells, indicating its antitumor potential.

Subsequently, computational analyses identified​ the STAT3/p53/SLC7A11 axis as a key regulatory pathway linked to ferroptosis in CRC.​ Network pharmacology identified 78 candidate targets, among which TP53 ranked first, with STAT3 and IL-6 also ranking among the top nodes. KEGG enrichment showed that “Pathways in cancer” and “Ferroptosis” were prominently enriched, suggesting a potential mechanistic link to ferroptosis regulation. Molecular docking and dynamics simulations further supported the interaction between bufothionine and these targets, guiding the selection of p53, STAT3, and SLC7A11 for experimental validation.

Guided by computational predictions, we designed experiments to verify if bufothionine induces ferroptosis-associated cell death. Our results suggest that bufothionine treatment in HT-29 cells led to significant accumulation of Fe^2+^, elevation of reactive oxygen species (ROS) and lipid peroxidation products (MDA), and depletion of reduced glutathione (GSH). These changes could be partially reversed by the ferroptosis inhibitor Fer-1, indicating that other forms of cell death, such as apoptosis, may also be involved. Mechanistically, Western blot showed bufothionine downregulated p-STAT3, SLC7A11, and GPX4. SLC7A11 is frequently overexpressed in CRC as a metabolic adaptation to oxidative stress, rendering cancer cells highly dependent on glucose and glutamine metabolism as well as GSH-based antioxidant defense; its suppression by bufothionine disrupts GSH synthesis and impairs GPX4 activity, thereby disabling the primary cellular defense against lipid peroxidation and sensitizing cells to ferroptosis-associated cell death (Xue et al., 2025; Liu et al., 2025; Cao et al., 2025; Koppula et al., 2021). Notably, Ferroptotic defense involves not only the GSH/GPX4 system but also mitochondrial pathways such as DHODH-mediated CoQ reduction, which suppresses mitochondrial lipid peroxidation and broadens our understanding of ferroptosis regulation in CRC (Mao et al., 2021). Bufothionine also significantly reduced mutant p53 protein levels in HT-29 cells. HT-29 cells predominantly express a mutant p53 protein, a feature commonly observed in many carcinoma cell lines (Midgley and Lane, 1997; Haupt et al., 1997; Azeez et al., 2025; Al-Keilani et al., 2018). Although mutant p53 loses its tumor suppressor function and fails to promote ferroptosis, its downregulation or degradation can restore a wild-type-like p53 function that promotes ferroptosis via the SLC7A11/GSH/GPX4 axis (Wang et al., 2026; Wu et al., 2026). Therefore, bufothionine-induced downregulation of mutant p53 may be associated with a diminished ferroptosis-suppressive effect, leading to restored ferroptotic sensitivity and tumor growth inhibition. This interpretation is supported by the concomitant downregulation of SLC7A11 and GPX4, accumulation of Fe^2+^ and lipid peroxides, and partial rescue by Fer-1. Additionally, bufothionine suppressed inflammatory mediators NLRP3 and IL-1β, potentially further relieving ferroptosis inhibition. Given the emerging interplay between iron metabolism and inflammation in regulating ferroptosis (Mo et al., 2024), this anti-inflammatory effect may further alleviate ferroptosis inhibition and reinforce the antitumor activity of bufothionine.

Finally, the anti-tumor efficacy and the proposed ferroptosis mechanism were further validated in an HT-29 xenograft mouse model. Bufothionine treatment significantly slowed tumor growth and alleviated pathological morphological changes, supporting its *in vivo* antitumor activity. Importantly, these effects were achieved without compromising systemic safety. Unlike 5-Fu, which induced marked clinical toxicity, bufothionine exhibited a favorable tolerability profile. Moreover, bufothionine improved liver function by attenuating tumor-induced elevations in AST and ALP without causing hepatosplenic toxicity, which is consistent with our previous findings (Xie et al., 2015; Kong et al., 2021). These results highlight its advantage over conventional chemotherapy. Recent studies have also reported that natural product-derived compounds show both good efficacy and a favorable safety profile (Cen et al., 2025; Wang Z. et al., 2024), suggesting that they represent an important source for the discovery and development of anticancer agents.

In addition,​ Systemic safety assessments indicated no significant hepatosplenic toxicity. Notably, bufothionine also favorably modulated the tumor cytokine profile: it downregulated IL-6, IL-10, and TGF-β, which are known to foster an immunosuppressive and pro-tumorigenic microenvironment in CRC (Thuya et al., 2025; Nguyen et al., 2020; Bandi et al., 2025). Conversely, it upregulated IFN-γ and TNF-α, two key cytokines that mediate anti-tumor immune responses (Hoekstra et al., 2024).

Mechanistically, analysis of tumor tissues confirmed that bufothionine downregulated p-STAT3, p53, SLC7A11, and GPX4, consistent with the modulation observed *in vitro*, and supporting the relevance of the STAT3/p53/SLC7A11 axis *in vivo*. Notably, this mechanism is closely linked to a key regulatory pathway in the TME.​ The IL-6/STAT3 axis is a known negative regulator of ferroptosis (Thuya et al., 2025), and its inhibition by bufothionine aligns with established strategies to target this pathway in CRC, as evidenced by studies showing that STAT3 inhibition can overcome therapeutic resistance in TP53-mutant models (Xin et al., 2025).

**FIGURE 8:**
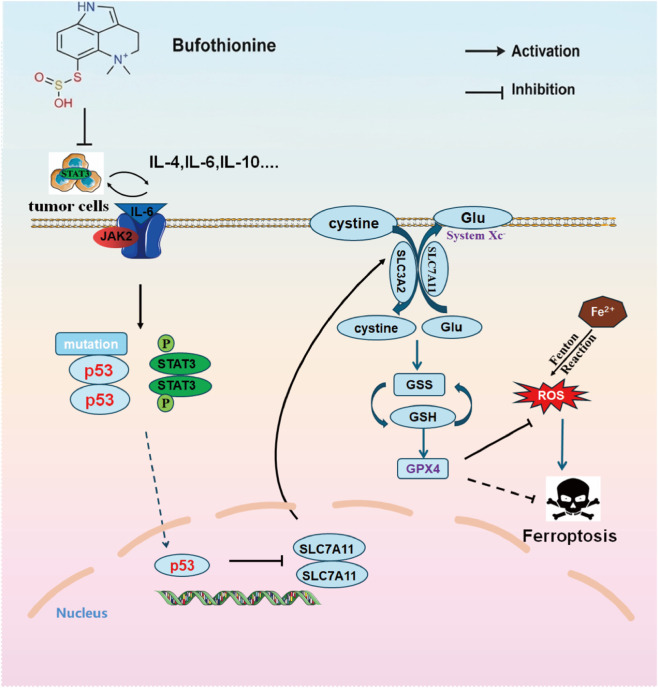
Signal pathway diagram of bufothionine in the treatment of colorectal cancer.

Collectively, our findings delineate an integrated mechanistic framework, as illustrated in Figure 8. Bufothionine suppresses IL-6-driven STAT3 activation and reduces mutant p53 levels, thereby downregulating SLC7A11, impairing cystine uptake and glutathione synthesis, inactivating GPX4, and ultimately sensitizing cells to lipid peroxidation and iron-dependent cell death. These results provide a mechanistic basis for the anti-tumor efficacy of bufothionine and highlight the STAT3/p53/SLC7A11 signaling axis as a viable target for CRC therapy.

Certain limitations of the present study should be acknowledged, and future investigations may be pursued to provide more robust evidence for its further development as an antitumor agent. These include (1) systematic direct binding assays (e.g., CETSA, SPR, DARTS): to experimentally validate the interaction between bufothionine and its predicted targets; (2) evaluation across multiple cancer cell lines with different p53 statuses​ to consolidate the reliability of its antitumor effects; (3) exploration of additional candidate targets identified by network pharmacology to elucidate its multitarget anticancer profile; and (4) in-depth investigation of the mechanisms underlying bufothionine-induced cell death beyond ferroptosis, such as apoptosis. Together, these efforts will offer a stronger foundation for the future development of bufothionine as a potential​ anticancer agent.

## 5 Conclusion

Bufothionine suppresses colorectal cancer via the STAT3/p53/SLC7A11 signaling axis in a manner linked to ferroptosis regulation, accompanied by modulation of the inflammatory microenvironment. These findings suggest its potential as a therapeutic agent for colorectal cancer.

## Data Availability

The datasets presented in this study can be found in online repositories. The names of the repository/repositories and accession number(s) can be found in the article/supplementary material.
